# Drought, Deluge and Declines: The Impact of Precipitation Extremes on Amphibians in a Changing Climate 

**DOI:** 10.3390/biology2010399

**Published:** 2013-03-11

**Authors:** Susan C. Walls, William J. Barichivich, Mary E. Brown

**Affiliations:** 1 Southeast Ecological Science Center, U.S. Geological Survey, 7920 NW 71^st^ Street, Gainesville, FL 32653, USA; E-Mail: wbarichivich@usgs.gov; 2 Cherokee Nation Technology Solutions, Contracted to U.S. Geological Survey, Southeast Ecological Science Center, 7920 NW 71st Street, Gainesville, FL 32653, USA; E-Mail: mbrown@usgs.gov

**Keywords:** *Ambystoma talpoideum*, amphibians, climate change, drought, flooding, Mole Salamander, occupancy, precipitation, rainfall pulses, southeastern United States

## Abstract

The Class Amphibia is one of the most severely impacted taxa in an on-going global biodiversity crisis. Because amphibian reproduction is tightly associated with the presence of water, climatic changes that affect water availability pose a particularly menacing threat to both aquatic and terrestrial-breeding amphibians. We explore the impacts that one facet of climate change—that of extreme variation in precipitation—may have on amphibians. This variation is manifested principally as increases in the incidence and severity of both drought and major storm events. We stress the need to consider not only total precipitation amounts but also the pattern and timing of rainfall events. Such rainfall “pulses” are likely to become increasingly more influential on amphibians, especially in relation to seasonal reproduction. Changes in reproductive phenology can strongly influence the outcome of competitive and predatory interactions, thus potentially altering community dynamics in assemblages of co-existing species. We present a conceptual model to illustrate possible landscape and metapopulation consequences of alternative climate change scenarios for pond-breeding amphibians, using the Mole Salamander, *Ambystoma talpoideum*, as an example*.* Although amphibians have evolved a variety of life history strategies that enable them to cope with environmental uncertainty, it is unclear whether adaptations can keep pace with the escalating rate of climate change. Climate change, especially in combination with other stressors, is a daunting challenge for the persistence of amphibians and, thus, the conservation of global biodiversity.

## 1. Introduction

Climate change is anticipated to be one of the most significant drivers of environmental change in the forthcoming century and, in combination with the spread of invasive species, habitat loss and fragmentation, emerging diseases and numerous other stressors, poses a formidable threat to global biodiversity [1,2,3,4,5,6,7]. Such threats are contributing to population declines of many organisms, as well as the loss of species, at unprecedented rates around the world [8]. This “biodiversity crisis” is thought to be indicative of a 6^th^ major extinction event [9], and global climate change is considered one of the main contributors to these extinctions [10]. The Class Amphibia is being especially affected, as it is experiencing more severe losses (currently estimated at nearly 40% of all species [6]) than any other taxonomic group studied [9,11]. Amphibians are useful as a model system for studying the impacts of a changing climate because amphibian reproduction is tightly tied to water quality, water availability, and patterns of rainfall, all of which are projected to be affected by climate change [12]. Moreover, individual species within a community vary greatly in their hydrologic preferences [13], which have implications for the effects of climate change on community composition. Thus, climate-induced changes in hydrology are potentially one of the biggest threats to most aquatic-breeding amphibians.

According to the Intergovernmental Panel on Climate Change (IPCC) [14,15], globally, the future risk of both floods and droughts will increase in a warmer climate. Model projections for the 2090s indicate that the proportion of the global land surface in extreme drought is predicted to increase by a factor of 10 to 30 [14,16]. The number of extreme drought events per 100 years and mean drought duration are anticipated to increase by factors of two and six, respectively, by the 2090s [14,16]. Simultaneously, the frequency of heavy rainfall or the proportion of total precipitation from heavy rainfall events will likely increase over many areas of the world in the 21^st^ century [15]. Such extreme climatic events are known to drive population “booms” [17], alter population and community-level interactions and, therefore, provide valuable insight into the processes that structure communities [18,19].

We explore the potential impacts of extreme precipitation events on amphibian populations. In particular, we highlight the effects of drought on the Mole Salamander, *Ambystoma talpoideum*, a pond-breeding amphibian from the southeastern United States. We use this species to represent ecologically-similar amphibians that are associated with freshwater habitats and pose hypotheses about the landscape and metapopulation-level consequences of variation in precipitation for this faunal group.

## 2. Overview of Climate Change Effects on Amphibians and Other Herpetofauna

Climate change is a likely factor in declines of amphibians and other herpetofauna in various parts of the world, including Australia [20], the Neotropics [21,22,23,24] (but see [25]), and the southeastern United States [26]. Rapid global warming poses a foreboding threat to reptiles with temperature-dependent sex determination, as shifts in temperatures will likely skew sex ratios, leading to demographic collapse [27]. The distributions and breeding phenologies of numerous species of herpetofauna are being altered around the world [8,28,29,30,31,32,33,34,35,36,37,38,39] (but see [40]). Parmesan [32] demonstrated that amphibians have shifted toward significantly earlier breeding—more than any other taxonomic/functional group—seasonally advancing more than twice as fast as trees, birds and butterflies. Climatic variation has been implicated in changes in body size/condition and reproduction in some frogs [41,42]. Climate-induced changes in geographic ranges have also been observed: three species of anurans in the tropical Peruvian Andes have colonized recently deglaciated habitats at record elevations for amphibians worldwide [8,33] and, in Spain, 29 species of reptiles have expanded their northern ranges in response to increases in temperature during the 20^th^ century [43]. Last, Raxworthy *et al.* [44] reported a trend of upslope movements in the distributions of montane amphibians and reptiles in Madagascar: over a 10-year period, these authors documented overall mean shifts in the elevational midpoint of 19–51 m upslope for 30 species representing five families of reptiles and amphibians. These upslope shifts are consistent with predictions of climatic warming. Climate-related environmental change may also contribute to movement of some hybrid zones [45]. In the southern Appalachian Mountains of the U.S., the upward spread of a hybrid zone between two terrestrial salamanders (*Plethodon teyahalee* and *P. shermani*) is correlated with increasing air temperatures, but not precipitation, over a 16-year period, suggesting that factors associated with a changing climate may have influenced this hybrid zone movement [46]. These studies mostly illustrate responses of herpetofauna to changes in temperature. Drought and flooding (from catastrophic storms and anthropogenic habitat alteration) likewise impact a variety of ecological, life history and population traits [47,48,49,50,51,52,53,54,55,56,57,58]. The impacts of such precipitation extremes on amphibian populations, in particular, are explored hereafter.

### 2.1. Effects of Drought on Amphibians

The IPCC considered drought to be either meteorological (precipitation well below average), hydrological (low river flows and water levels in rivers, lakes and groundwater), agricultural (low soil moisture), or environmental (a combination of the above) [14]. For the purposes of our review, we discuss the impact any of these types of drought have on amphibians.

All amphibians depend to some extent on the availability of fresh water for successful reproduction, regardless of whether they engage in direct development in the terrestrial environment or deposit their eggs in aquatic habitats [59]. Soil moisture availability is a vital resource for terrestrial-breeding species with direct development, such as many lungless salamanders [60,61]. For these species, the risk of evaporative water loss is likely the most important constraint on embryonic survival. In 1970, one population of an endemic and federally endangered terrestrial salamander (the Shenandoah Salamander, *Plethodon shenandoah*) was extirpated due to a short-term drought, coupled with interspecific competition with the Eastern Red-backed Salamander (*P. cinereus*) [61]. Over a 14-year period, a population of another terrestrial amphibian with direct development, the Puerto Rican Coquí (*Eleutherodactylus coqui*), declined in response to an increase in the duration and frequency of periods without rain, with the abundance of frogs in a given year inversely proportional to the longest dry periods during the previous year [62]. Similarly, Burrowes *et al.* [24] documented an association between years with extended periods of drought and the decline of eight species of *Eleutherodactylus* in Puerto Rico over a 12-year period. Juvenile *Eleutherodactylus* are likely unable to survive extensive drought, and the potential risk of desiccation may affect adult foraging during extended dry periods [62].

Amphibians that breed in temporary, vernal pools and intermittent headwater streams are also susceptible to fluctuations in temperature and precipitation, as evapotranspiration losses could possibly exceed precipitation during cyclical droughts, resulting in drying of aquatic sites [63,64,65,66]. Insufficient rainfall, extreme drought and/or shortened hydroperiods have been linked with (1) declines in anuran calling activity [20,67]; (2) catastrophic reproductive failure in numerous pond-breeding amphibians [68,69,70,71,72,73,74]; (3) metamorphosis at smaller body sizes [75,76], (4) the potential local elimination of paedomorphosis in salamanders [75], and (5) local extinctions [20]. As much as 90% of a population of the Mole Salamander (*Ambystoma talpoideum*) may skip breeding in a drought year [77], lowering the reproductive output of that population in such years. Similarly, breeding probabilities for female Eastern Tiger Salamanders (*A. tigrinum*) may be reduced by more than 50% in drought years [78]. Such climate-induced complete or partial reproductive failure is a likely contributor to population declines in many species of amphibians [26,74,79].

In addition to its effects on survival, reproduction and juvenile recruitment, prolonged periods of drought may affect occurrence of amphibians across a landscape, as well as estimates of extinction and colonization, which drive changes in occupancy and the metapopulation dynamics of species within a region. For example, in Michigan, U.S., severe drought affected two syntopic anuran species (the Spring Peeper, *Pseudacris crucifer* and the Western Chorus Frog, *P. triseriata*) in different ways: drought reduced pond hydroperiods and densities of aquatic predators which, for the chorus frog, facilitated colonization of 15 new ponds and exponential growth in regional population size (and, thus, decreased extinction probability) [80]. In contrast, colonization probability for the Spring Peeper remained relatively constant over the 11-year period of study, but drought altered which ponds were suitable as sources for metapopulation persistence [80]. In a 13-year monitoring effort in southern Australia, the most severe drought on record negatively affected the probability of site occupancy by the endangered Northern Corroboree Frog (*Pseudophryne pengilleyi*) [20]. For this species, 42% of breeding sites became unsuitable due to fewer pools with less water and drying-related tree encroachment into ponds [20].

In another long-term study (14 years), a severe, prolonged drought reduced annual occurrence of Wood Frog tadpoles (*Lithobates sylvaticus*) in individual pools over five consecutive years throughout central Saskatchewan, Canada, but had no observable long-term effect on either tadpole occupancy or abundance [81]. Similarly, Price *et al.* [82] documented that larval occupancy of the Northern Dusky Salamander (*Desmognathus fuscus*) in first-order streams of North Carolina, U.S., decreased by 30%, on average, during a prolonged drought. Survival of adult salamanders was relatively high and adult occupancy remained stable over the five-year study, although temporary emigration probabilities doubled during the drought period [82]. These authors suggested that high survival of adult *D. fuscus*, coupled with their temporary emigration, may compensate for the negative effects of drought on larvae and facilitate resiliency of this species to drought conditions.

### 2.2. Effects of Deluge from Major Storm Events on Amphibians

A population decline in another stream-dwelling salamander (the Spring Salamander, *Gyrinophilus porphyriticus*) has been attributed to increased precipitation (leading to stream flooding and high-velocity water flow) associated with climate change in northeastern North America [83]. Lowe [83] suggested that mortality of metamorphosing individuals is high during spring and fall floods, which have increased in volume and frequency with increasing precipitation in this region. Consequently, adult recruitment in this population declined significantly over a 12-year period, with no trend in larval abundance. Flooding, with its associated high water flow and transport of debris (sediment, boulders, large sections of wood and other vegetation) has been linked to declines and extirpations of a variety of other stream and river-dwelling amphibians as well [84,85,86] (but see [87]). For a pond-breeding amphibian, hurricane-related flooding from heavy rainfall prevented breeding in a population of the terrestrial Marbled Salamander (*Ambystoma opacum*) in North Carolina [88]. In autumn, females of this species migrate to the dry basins of temporary woodland ponds, which later fill from winter rains, to deposit and then brood their egg clutches [89]. Pond-filling stimulates well-developed larvae to hatch, at which time females emigrate from breeding sites and return to terrestrial refugia [89]. Premature filling of ephemeral ponds can force females to oviposit along the outer margins of the pond basin, which may not be inundated later in the season; can cause mortality of embryos in the pond basin [90], or prevent reproduction altogether. Thus, flooding of breeding sites from late-season hurricanes can have catastrophic effects on the nesting success of this terrestrial-breeding species. With respect to reproductive phenology, the timing (in addition to severity) of hurricane events contributes to the magnitude of a storm’s effect on other organisms as well [91].

In the Caribbean, hurricanes and other tropical cyclones have impacted a variety of taxa, including amphibians, in complex ways [18,91]). Abundance of the Puerto Rican Coquí, *Eleutherodactylus coqui*, increased following two hurricane events (Hurricanes Hugo and Georges) that impacted Puerto Rico in 1989 and 1998, respectively [92,93,94]. In contrast, relative abundances of two other species of *Eleutherodactylus* were significantly lower following Hurricane Georges, and overall species richness and evenness declined as well [94]. The increased abundance of *E. coqui* following Hurricane Hugo was attributed to a decrease in abundance of invertebrate predators, coupled with the presence of downed canopy debris on the forest floor, which provided quality retreat sites for colonization [93]. Hurricane-related disturbance of the forest canopy also allowed establishment of vegetation that provided preferred frog nesting sites [93].

Isolated coastal wetlands may be exposed to saline waters as a result of storm surge during hurricane events, such as those associated with four hurricanes that hit the Gulf Coast of the U.S. in 2004 and 2005 [95,96]. In 2005, storm surge overwash from Hurricane Dennis had no long-term effect on amphibian species richness at a coastal site in the panhandle region of Florida, U.S. [96] (but see Schriever *et al.* [95] for effects of hurricanes on amphibian community composition in other geographic regions). Some amphibians may be locally adapted [97,98] to rapid changes in salinity and other water chemistry parameters, which occur during brief intervals of flooding from salt water intrusion during hurricanes [96]. Brown and Walls [99] documented that species of anuran amphibians commonly associated with coastal freshwater wetlands differ in their salinity tolerances, suggesting that salt water intrusion due to storm surges and sea level rise may affect species composition of these ecosystems. Moreover, climate change, via encroaching sea level rise and perturbations from hurricane-related saltwater intrusion, may also indirectly facilitate the spread of non-indigenous species (such as the Cuban Treefrog, *Osteopilus septentrionalis*) that have a higher tolerance of saline habitats than do native species [99].

The frequency of tropical storms and major hurricanes in the North Atlantic has increased over the past 100 years [15]. Under global warming scenarios for the 21^st^ century, current climate models and downscaling techniques consistently project increases in intensity and the number of more intense storms, along with increases in tropical cyclone-related rates of rainfall [15]. For example, the intensity of the hybrid “superstorm” Hurricane Sandy, which devastated the northeastern U.S. in late October 2012, was likely exacerbated by the excessively warm waters off New England at that time (1.3 °C above average in September, the second-warmest September in recorded history http://www.wunderground.com/blog/JeffMasters/comment.html?entrynum=2276). Globally, an overall decrease or no change in the frequency of tropical cyclones in the 21^st^ century is expected [15]. Current climate models project a 28% reduction in the overall frequency of Atlantic storms, yet an 80% increase in the frequency of Saffir-Simpson category 4 and 5 Atlantic hurricanes over the next 80 years under the A1B emissions scenario [15]. Although amphibians in temperate and tropical regions have likely evolved behaviors and life histories in response to cyclonic storms and other forms of environmental uncertainty, it is questionable whether amphibians and other organisms will be able to keep pace with the current, escalating rate of environmental change [100,101,102,103,104,105].

### 2.3. Rainfall Pulses versus Total Amount of Precipitation

Rainfall is an important stimulus for reproduction in many pond-breeding amphibians [68,77,106,107,108,109,110]. Since 1910, there has been approximately a 10% increase in precipitation across the contiguous United States [111], and is expected to continue to increase in many areas during the next century [12]. This increase is partly due to an increase in the frequency of extremely heavy precipitation events, characterized by “pulses” of intense, heavy rain, separated by longer dry intervals [111,112]. In the U.S. and elsewhere, the proportion of total precipitation derived from extreme, heavy events is increasing relative to more moderate rainfall episodes [17,111,112]. Burkett and Kusler [113] proposed that changes in precipitation patterns (not simply total precipitation) may have significant impacts on wetland-dependent species. Climate change models predict the occurrence of more variable patterns of precipitation, with longer droughts and larger (but fewer) rainfall events, in addition to increased temperatures [114,115]. Such variation in the temporal distribution, rather than the total amount of rainfall per se may be an important correlate of population fluctuations in amphibians [62].

To our knowledge, the potential effects of variable patterns of precipitation, with extended droughts and fewer, yet larger rainfall events, have largely been overlooked in studies with amphibians (but see [116,117]). Instead, studies with amphibians have focused almost exclusively on the effects of increased temperature and total precipitation. There is a need to test the hypothesis that the pattern and timing of rainfall events, rather than total amount of rainfall, may become increasingly more influential on amphibians, especially in relation to seasonal reproduction. Data collected over many years in a long-term monitoring program will be necessary to address this hypothesis, at least with relatively rare or otherwise infrequently detected species.

## 3. Landscape and Metapopulation-level Effects of Extreme Climatic Events: An Example with the Mole Salamander, *Ambystoma talpoideum*

Climatic factors may have a profound impact on amphibian populations and communities at multiple spatial and ecological scales [80,118]. At the landscape level, climate-driven shifts in pond hydroperiods can alter habitat (breeding site) heterogeneity, a necessity for persistence of diverse communities [80]. Landscape features (e.g., landcover type, numbers, sizes, and spatial relationships of wetlands) may act as drivers of metapopulation dynamics, altering connectivity among sites, extent of genetic isolation, and rates of extinction and colonization [119,120,121,122].

**Figure 1:**
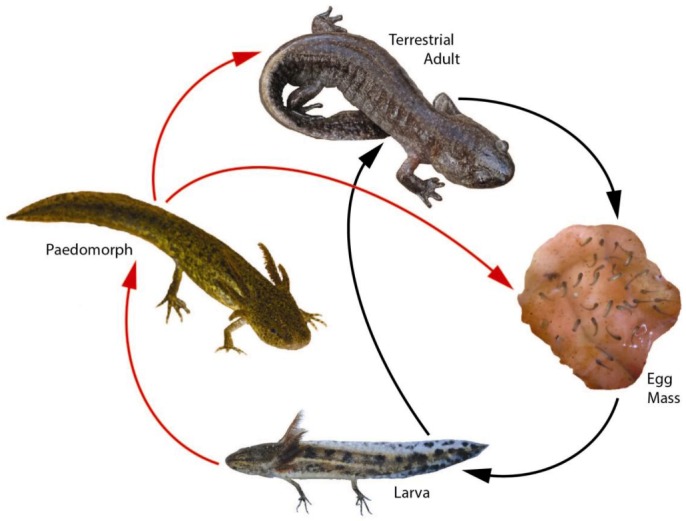
Complex life cycle [132] of the Mole Salamander, *Ambystoma talpoideum*. Black arrows indicate metamorphic pathway; red arrows indicate paedomorphic pathway. Except during breeding periods, metamorphosed adults occur in the terrestrial habitat; all other life stages are aquatic.

Many populations of pond-breeding amphibians may exist as metapopulations, depending on the extent to which their habitat is fragmented [123]. Some ambystomatid salamanders satisfy the conditions necessary to qualify as metapopulations, but conclusions vary depending on the type of data used to estimate dispersal ([124], and references therein). The Mole Salamander (*Ambystoma talpoideum*) is a pond-breeding amphibian that occurs throughout much of the Coastal Plain of the southeastern U.S., ranging from South Carolina to eastern Texas and northward to southern Illinois (with disjunct populations occurring outside this region) [89]. Based on genetic data, one South Carolina population of this species does not appear to operate in a metapopulation context [125], although the extent to which populations elsewhere in this species’ range may exist as metapopulations is not known. The Mole Salamander is typically associated with fishless, seasonal wetlands, although this species has been found naturally-occurring with fish elsewhere on the Atlantic Coastal Plain of the U.S. [126]. Individuals of this species can be facultatively paedomorphic (*i.e.*, become sexually mature in the aquatic environment while retaining larval features; Figure 1) in fish-free ponds with long hydroperiods [126]. Alternatively, aquatic larvae are capable of metamorphosing in 4–5 months in landscapes with temporary ponds that dry annually [26,106], resulting in populations that consist of predominantly metamorphosed, terrestrial adults (Figure 1). Paedomorphic adults (Figure 1) predominate in permanent and semi-permanent ponds, where they can persist 14–15 months [127]. Pond drying influences the expression of these alternative life history strategies, although its propensity to do so varies among populations [128].

We conduct long-term, on-going monitoring in the panhandle region of northwest Florida on populations of aquatic larval and paedomorphic *A. talpoideum*. We use an information-theoretic, model-selection framework to detect patterns in site occupancy [129] and changes in trends over time, especially as they relate to changes in climate. Seasonal estimates of occupancy, corrected for imperfect detection, declined from 22.3% of ponds in Spring 2009 to 9.9% of ponds in Fall 2012 [130]. Our best supported occupancy model suggested that changes in occupancy for larvae and paedomorphs were driven by increased rates of extinction (*i.e*., the probability that a site occupied in season *t* is unoccupied in season *t*+1) that corresponded with drought-related drying of ponds. Under a scenario of ongoing drought, local extinction increases and occurrence probabilities decrease as long-hydroperiod breeding sites dry prematurely [130].

The percentage of the Southeast experiencing moderate to severe drought has increased, yet many parts of this region have also experienced an increase in the occurrence of heavy downpours [131]. Climate models project that these patterns will persist in the future for the Gulf Coast states [131]. Based on our observations of drought-induced changes in site occupancy, along with the projection of continued variation in future climate for the Southeast, Walls *et al.* [130] predicted that increases in severity and occurrence of drought will likely result in shortened hydroperiods and an overall loss of long hydroperiod wetlands across the landscape: long hydroperiod wetlands will be reduced to ones of intermediate hydroperiod and existing short hydroperiod sites will likely dry completely, increasing the distances between those sites that persist. The loss of species adapted to long-hydroperiod habitats, along with the elimination of predatory fishes from such sites, will modify the composition of communities as well as the dynamics of competition and predation within those assemblages.

**Figure 2:**
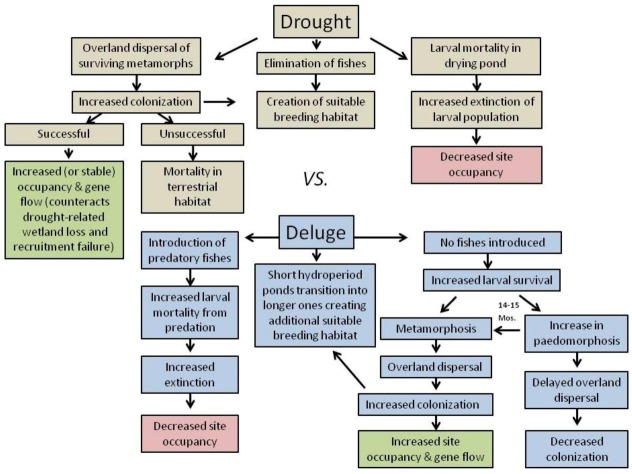
Hypothesized landscape and metapopulation-level consequences of alternative climate change scenarios (drought *versus* deluge) for the Mole Salamander, *Ambystoma talpoideum*. Consequences of decreased precipitation are shown in tan and those from increased precipitation are shown in blue. In both scenarios, site occupancy may either increase (in green) or decrease (in red).

Connectivity and linkages among local sites is needed for dispersal, colonization and persistence. Consequently, by increasing the distance among remaining sites, this drought-induced wetland loss may increase mortality of dispersing juveniles in the terrestrial habitat and negatively impact site occupancy, dispersal, colonization and, potentially, larger metapopulations and communities [133] (Figure 2). Pond-breeding amphibians are well-known for fidelity to their natal ponds [133] yet, for populations to persist, terrestrial juveniles and adults must disperse and successfully colonize newly created short hydroperiod ponds (formerly intermediate ones). Pond-drying may promote rapid larval growth, metamorphosis, and dispersal. Those metamorphs that successfully colonize new sites will contribute to gene flow among populations, and a predominantly paedomorphic population will transition to one composed of metamorphosed individuals (Figure 2).

Alternatively, an increase in precipitation may increase the number of wetlands on the landscape, including forming “new” wetlands that currently do not exist or have hydroperiods that are currently too short for successful amphibian reproduction [130]. Currently short-hydroperiod wetlands may disappear as all sites become persistently inundated, increasing local extinctions of amphibians adapted to ephemeral habitats [134,135,136]. During flood events, sites may be colonized by aquatic predators (e.g., fishes and aquatic insects), which would increase larval mortality from predation, increase local extinctions and, thus, decrease site occupancy (Figure 2). By losing “short hydroperiod” species [130] and introducing fishes into sites, once again the composition of communities, and the competitive and predatory interactions within them, will likely be altered. In the absence of predatory fish, the proportion of the population that follows a metamorphic pathway will disperse and colonize new sites, resulting in an overall increase in site occupancy and gene flow for metamorphs (Figure 2).

The availability of long hydroperiod ponds may therefore selectively favor the production of paedomorphic populations, decreasing overland dispersal and colonization of new sites, thus inhibiting gene flow within metapopulations (Figure 2). Petranka [137] similarly proposed that an increased time to metamorphosis—as would occur in long hydroperiod ponds—could increase the risk of metapopulation extinction in amphibians with complex life cycles. Moreover, because life history traits and metapopulation dynamics are linked, climate change and other stressors that affect metapopulations could also influence the evolution of life history traits [137].

Figure 2 illustrates several important points. (1) A comprehensive view of the effect of climate change on amphibians with complex life cycles requires monitoring both aquatic (larval and paedomorphic) and terrestrial (metamorphosed juvenile and adult) life history stages. (2) Site occupancy may either increase or decrease in both the drought and deluge scenarios, emphasizing the challenge of separating the effects of extremes in precipitation (increases in both drought and heavy rainfall events) in the same geographic region [111,112]. (3) The complexities of the pathways leading to similar occupancy outcomes illustrate the importance of identifying the mechanism(s) by which change in occupancy occurs. In summary, metapopulation dynamics, gene flow, and the transition between predominantly paedomorphic *versus* metamorphic populations may be strongly influenced by wetland hydrology and connectivity which, in turn, may be altered by climate change.

The Southeastern U.S. encompasses some of the richest biodiversity hotspots in North America and is heralded as a center of endemism for many taxa [138]. For amphibians, two families of salamanders (Amphiumidae and Sirenidae) are endemic to the Southeast, and 10 salamander genera have their centers of distribution within the Southeast [139]. This region also hosts the highest diversity of forested and freshwater aquatic habitats [140,141], as well as the richest aquatic fauna of any temperate area in the world [142]. Thus, in terms of biodiversity, this species-rich region potentially has much to lose due to climate change and other threats [143]. The Mole Salamander is ecologically similar to other pond-breeding amphibians, making it a useful “surrogate” for species that are of conservation concern. Predicting how relatively common species like the Mole Salamander respond to climate change is an important first step toward understanding how species of conservation concern may be affected.

## 4. Conclusions

Climate change—including extremes in precipitation—is forecast to be one of the most significant drivers of ecological change in the forthcoming century [3]. Changes in wetland hydrology are potentially one of the biggest threats to most aquatic-breeding amphibians. Variation in seasonal rainfall affects pond hydroperiods and the timing of amphibian reproduction, which may modify the composition of communities and interfere with the dynamics of competitive and predatory interactions within those assemblages. Some ecologically-similar species (e.g., stream-dwelling salamanders) can respond to precipitation extremes in contrasting ways [82,83], thus exacerbating the challenge of designing ecosystem-level management plans to counteract climate change. The severity of precipitation effects depends upon the species, its propensity for phenotypic plasticity [144], and the life history stage that is impacted. Thus, a complete understanding of climate change effects on amphibians with complex life cycles requires monitoring both aquatic (larval and paedomorphic) and terrestrial (metamorphosed juvenile and adult) life history stages. The short-term effects of drought and deluge can be catastrophic, yet the long-term consequences are known for only a few species that have been monitored continuously for many years. We emphasize that drought and the timing, frequency and pattern of precipitation may impact pond-breeding amphibians, yet such alternatives to the more traditional focus on increases in temperature and total precipitation as metrics of climate change have rarely been considered for amphibians.

Climate change will likely exacerbate the negative effects of habitat fragmentation on amphibian metapopulations by reducing the number of inundated wetlands during droughts, thus increasing dispersal distances among sites. In contrast, flooding from heavy precipitation events can mix aquatic larvae from neighboring sites and introduce fishes and other predators into normally isolated wetlands. Using the Mole Salamander as an example of a pond-breeding amphibian that is subjected to such environmental variation, we present a conceptual model that illustrates how climate-driven changes in site occupancy, extinction and colonization rates may impact metapopulations. For this species, metapopulation dynamics, gene flow, and the transition between predominantly paedomorphic *versus* metamorphic populations may be strongly influenced by wetland hydrology and connectivity which, in turn, may be altered by climate change. Our model further indicates that, for the Mole Salamander and ecologically-similar species, site occupancy may either increase or decrease under both drought and deluge scenarios. The complexity of these potential outcomes underscores the importance of identifying the mechanisms (e.g., changes in extinction and colonization) by which drought and deluge drive biotic and abiotic (wetland hydrology) dynamics. Evaluating the pathways by which climatic variation leads to ecological change helps to identify gaps in our understanding of how amphibians respond to a changing climate and reveals the challenges of mitigating for the loss of this biodiversity. 
